# Cross-modal noise compensation in audiovisual words

**DOI:** 10.1038/srep42055

**Published:** 2017-02-07

**Authors:** Martijn Baart, Blair C. Armstrong, Clara D. Martin, Ram Frost, Manuel Carreiras

**Affiliations:** 1 BCBL. Basque Center on Cognition, Brain and Language, Donostia - San Sebastián, Spain; 2 Department of Cognitive Neuropsychology, Tilburg University, Tilburg, The Netherlands; 3 Department of Psychology & Centre for French & Linguistics at Scarborough, University of Toronto, Toronto, Canada; 4 IKERBASQUE Basque Foundation for Science, Bilbao, Spain; 5 Department of Psychology, The Hebrew University of Jerusalem, Jerusalem, Israel; 6 Haskins Laboratories, New Haven, CT, USA; 7 University of the Basque Country. UPV/EHU, Bilbao, Spain

## Abstract

Perceiving linguistic input is vital for human functioning, but the process is complicated by the fact that the incoming signal is often degraded. However, humans can compensate for unimodal noise by relying on simultaneous sensory input from another modality. Here, we investigated noise-compensation for spoken and printed words in two experiments. In the first behavioral experiment, we observed that accuracy was modulated by reaction time, bias and sensitivity, but noise compensation could nevertheless be explained via accuracy differences when controlling for RT, bias and sensitivity. In the second experiment, we also measured Event Related Potentials (ERPs) and observed robust electrophysiological correlates of noise compensation starting at around 350 ms after stimulus onset, indicating that noise compensation is most prominent at lexical/semantic processing levels.

Information about external events in the world enters the system through our senses, but the input is often degraded. The system can overcome unisensory noise in the signal and stabilize the percept by integrating information from multiple senses (e.g., refs 1 and 2). This is also true for complex signals such as human speech, wherein noise in the auditory speech signal can be compensated for by simultaneously presented visual speech (i.e., the articulating mouth of a speaker, e.g., refs 3, 4, 5) or printed text (e.g., ref. 6). Intriguingly, this latter type of compensation occurs despite the fact that print-speech mappings emerged relatively recently in evolution, and the system has not been biologically tailored for this type of audiovisual (henceforth, AV) correspondence.

Noise compensation in speech and in print can be assessed with a “matching task” in which a written and a spoken word are presented simultaneously, with one or both inputs masked by noise, and participants have to indicate whether the same word (congruent trials) or different words (incongruent trials) were presented across the two modalities. The underlying rationale is that asking participants to detect AV correspondence requires one or more interaction(s) between the unisensory inputs, during which cross-modal compensation for noise can be instantiated. This task has been used as an alternative to lexical decision or naming tasks in which one cannot be certain whether participants’ responses are driven by the auditory/phonological or visual/orthographic aspects of the signal^6^. Likewise, when AV noise compensation is assessed by comparing noisy unimodal trials to AV trials where there is one noisy signal and one clear signal, one cannot rule out the possibility that the AV gain relative to unimodal trials is driven by the clear unimodal signal, rather than cross-modal noise compensation per se (except when AV stimuli generate percepts that are different from either A or V in isolation, see ref. 7).

Although the matching task has produced evidence for interactions between speech and print (see e.g., refs 6 and 8), the interpretation of correct “match” responses on AV congruent trials (in which one of the signals is masked by noise) is complicated by several factors. Firstly, it is well known that participants may trade-off response speed (i.e., reaction times, henceforth RT) for accuracy (e.g., ref. 9). Therefore, high/low proportions of correct “match” responses may be the result of (i) a general tendency to respond more quickly or more slowly, (ii) successful noise compensation, or (iii) a combination of both (assuming that noise compensation takes time, the degree of compensation could vary with RT). Secondly, the proportion of incorrect “match” responses (when responding “match” to AV incongruent trials) needs to be considered, as participants may adopt a bias to respond “match” or “mismatch” on all trials, irrespective of AV congruency. Thirdly, accuracy is related to the actual noise levels as participants will become more sensitive to the match/mismatch as the signal-to-noise ratio increases. In two experiments, we isolated noise compensation effects in words when controlling for RT, bias and sensitivity. This rigorous assessment approach allowed us to conclude that noise compensation can be explained by accuracy (Experiment 1), and mainly occurs on a lexical/semantic level of processing (Experiment 2).

To assess the effects of noise compensation more formally, we quantified this construct using the following logic: First, we assumed that performance on AV stimuli in the clear (henceforth No_noise_) reflects a base-line ability to detect correspondence of print and speech under optimal conditions. From this baseline, we subtracted the accuracy differences under unimodal noise conditions (i.e., A_noise_ and V_noise,_ for noise in the auditory and visual modality), and summed them. The resulting value was then subtracted from the accuracy detriment for bimodal noise (AV_noise_) relative to No_noise_ (i.e., noise compensation, or Acc_comp_ = (No_noise_ − AV_noise_) − [(No_noise_ − A_noise_) + (No_noise_ − V_noise_)]). This compound noise compensation score increases when the No_noise_ − A_noise_ and No_noise_ − V_noise_ differences become smaller (and the No_noise_ − AV_noise_ difference becomes larger). In Experiment 1 participants were asked to respond as quickly as possible and we assessed the relationship between these accuracy differences relative to No_noise_ and noise compensation, when taking into account the interplay between accuracy, RT, bias, and sensitivity.

Experiment 2 was a non-speeded variant of the first task in which we probed the time-course of noise compensation via Event-Related Potentials (ERPs). This allowed us to disentangle the noise compensation process from processes related to bias and sensitivity in post-lexical response processes. Although it is debated whether orthography and phonology interact at all (e.g., ref. 10), there is also evidence for interactions occurring at a (sub) lexical level (e.g., refs 6, 11 and 12). As highlighted above, we assume that noise compensation has to be instantiated during at least one such interaction, and the time-course of the neural correlates that underlie noise compensation would reveal at which level(s) of processing noise compensation occurs.

To preview the main results, noise compensation increased when the difference between the unimodal noise conditions and the No_noise_ condition decreased, which persisted when RT, bias and sensitivity were controlled for. Furthermore, in a 350–390 ms window after stimulus onset, the ERP difference waves of the unimodal noise conditions relative to No_noise_ were not modulated by bias or sensitivity, but could explain noise compensation in accuracy. This result indicated that that compensation for noise in speech and print predominantly occurs at a lexical/semantic level of processing.

## Results

### Behavioral

**Figure 1:**
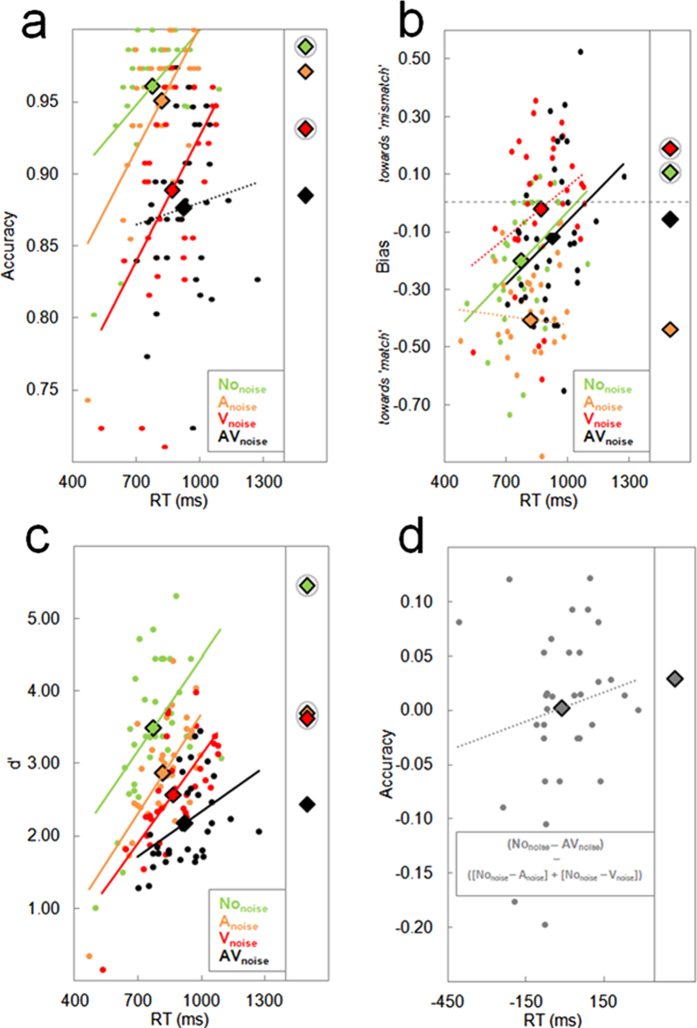
Scatter plots, linear trends and group averages between RT and accuracy (panel a), bias (panel b), sensitivity (panel c) and Acc_comp_ (panel d). The individual data (dots) and linear trends are data from Experiment 1, with group averages represented by diamonds. The narrow plots on the right of each panel represent the averages in Experiment 2 (where RT was not informative given the non-speeded nature of the task). All linear trends between RT and accuracy were positive, except for A_noise_ bias. Dotted lines indicate that linear trends were not significant, and grey circles indicate significant differences between group averages in Experiments 1 and 2.

First, we explored how accuracy, bias, sensitivity and accuracy-based noise compensation were modulated by RT, using the noise compensation score described in the introduction (i.e., Acc_comp_: [No_noise_ − AV_noise_] − ([No_noise_ − A_noise_] + [No_noise_ − V_noise_]). Based on signal detection theory, we quantified sensitivity through d-prime (d′) and used response criterion *c* as a measure of response bias (e.g., refs 13 and 14). As illustrated in Fig. 1, the linear trends between RT and accuracy, bias, sensitivity and Acc_comp_ values in the speeded task (Experiment 1) always carried over to the non-speeded task (Experiment 2). This is reflected by the fact that averages in Experiment 2 increased/decreased relative to Experiment 1 (although differences between were not always significant), according to the positive/negative linear relationships related to RT.

Because of the way we defined the compound Acc_comp_ score, it should increase when accuracy differences between A_noise_/V_noise_ and No_noise_ become smaller, and conversely, when unimodal noise detriments are high, Acc_comp_ is low. Before assessing this however, we first determined how accuracy related to bias and sensitivity. To do so, we correlated accuracy with bias and sensitivity for each noise condition separately, which was preferred over multiple linear regression because of multicollinearity between the predictors. In both experiments, bias and sensitivity were highly and significantly correlated with accuracy in most conditions: accuracy increased with a decrease in bias, and an increase in sensitivity. This finding also held for the partial correlations in which we controlled for RT (in Experiment 1 only, see Table 1).

Taken together, it is clear that effects of RT, bias and sensitivity cannot be ignored when explaining Acc_comp_ through accuracy. Therefore, the correlations between Acc_comp_ and the A_noise_, V_noise_ and AV_noise_ accuracy differences relative to No_noise_ were also computed when controlling for bias and sensitivity in all conditions (the last column in Table 1). These partial correlations showed that in both experiments, noise compensation increased when the unimodal noise accuracy detriments relative to No_noise_ decreased. This was not the case for the No_noise_ − AV_noise_ accuracy difference, as it was not significantly correlated with Acc_comp_ in Experiment 1, and was negatively correlated with Acc_comp_ in Experiment 2 (i.e., the smaller the difference between AV_noise_ accuracy and No_noise_ accuracy, the larger Acc_comp_). The partial correlations in Experiment 2 were also clearly stronger than the uncontrolled correlations, which suggests that in the non-speeded task, processes related to bias and sensitivity somehow interfered with noise compensation. It is therefore likely that processing of noise compensation, bias and sensitivity overlap in time (at least partially). This was assessed empirically in the ERP data.

### ERPs

**Figure 2:**
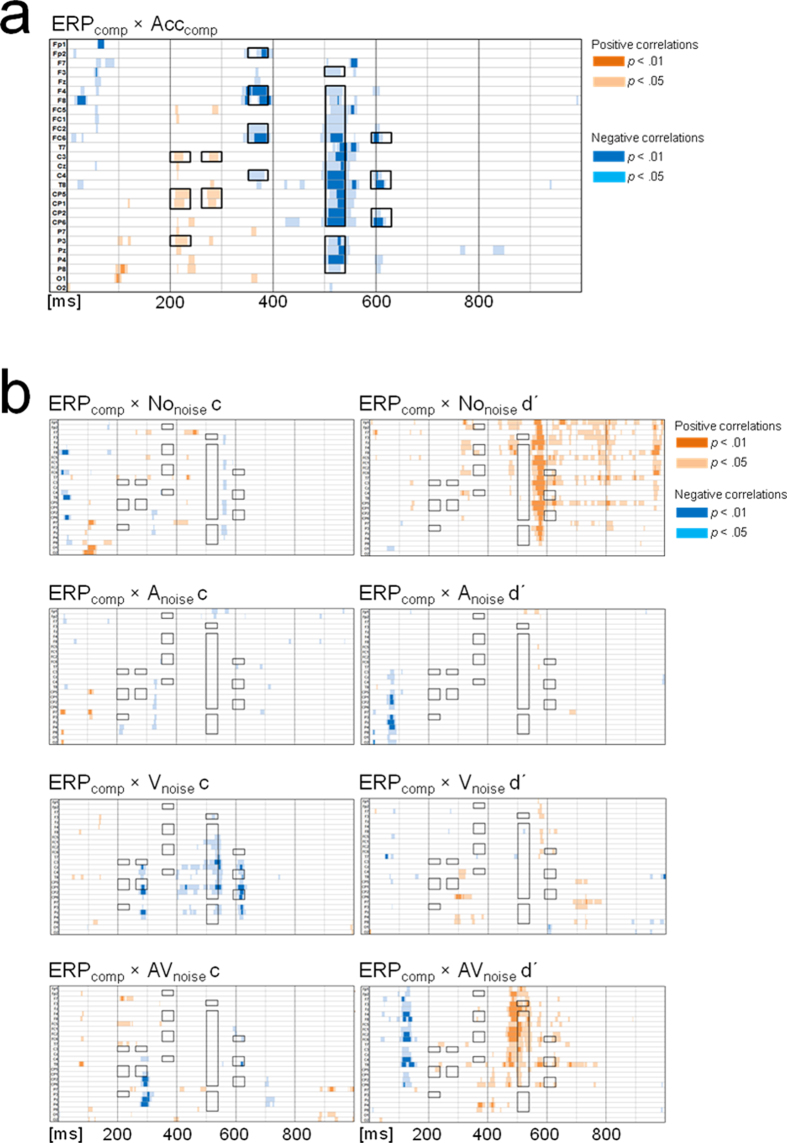
Correlations between ERP_comp_ and Acc_comp_ (panel a), between ERP_comp_ and bias (criterion c; panel b, left plots) and between ERP_comp_ and sensitivity (d′; panel b, right plots). The black outlines represent the clusters of electrodes in five 40-ms time-windows where ERP_comp_ and Acc_comp_ correlated in at least three electrodes (i.e., 200–240 ms, 260–300 ms, 350–390 ms, 500–540 ms, 590–630 ms).

To determine the neural correlates of Acc_comp_, we constructed the ERP_comp_ difference wave (using the same formula described previously, see Supplementary Information for an expanded rationale) and correlated this waveform with the behavioral Acc_comp_ measure (see Fig. 2a). Based on this exploratory correlation analysis, we selected five 40-ms time-windows in which ERP_comp_ correlated with Acc_comp_ in at least three electrodes that formed a spatial cluster. As illustrated in Fig. 2a, these criteria included all correlations that coincided in time and topography, and were purposely chosen to include all potentially relevant spatial-temporal clusters.

Next, we correlated ERP_comp_ with bias and sensitivity in all conditions. Figure 2b plots these relationships and shows that there was temporal-spatial overlap between these correlations and those between ERP_comp_ and Acc_comp_ (see for example the 500–540 ms window).

**Figure 3:**
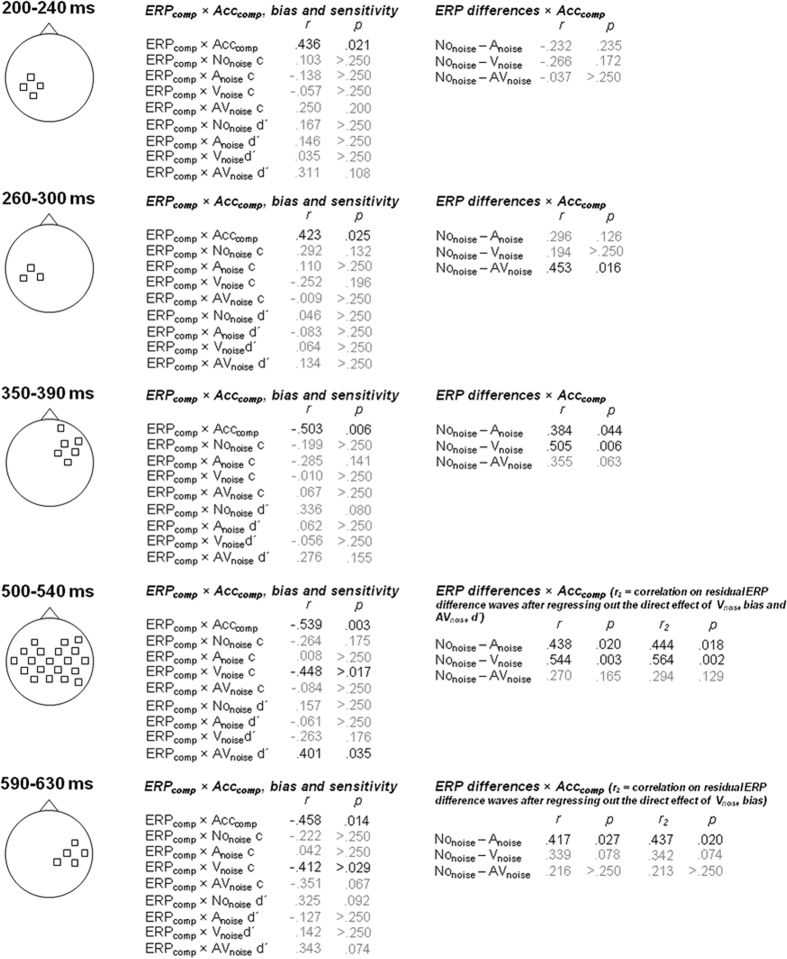
Correlations between ERP_comp_ averaged across electrodes (electrode sites correspond to Fig.2 and are indicated on the scalp) and Acc_comp_, bias (criterion c) and sensitivity (d′) in 40 ms windows of averaged activity (i.e., 200–240 ms, 260–300 ms, 350–390 ms, 500–540 ms, 590–630 ms). The right panels show the correlations between the ERP difference waves (relative to Nonoise) and Acc_comp_, in which the direct effects of V_noise_ bias (500–540 ms, and 590–630 ms) and AV_noise_ sensitivity (500–540 ms) were regressed out.

Next, we computed the ERP_comp_ averages (over both time and electrodes) for each of the temporal spatial clusters, and correlated the averaged ERP_comp_ with Acc_comp_, bias, and sensitivity. The results of these analyses are summarized in Fig. 3. Because the temporal-spatial clusters were selected based on the ERP_comp_ × ACC_comp_ correlations, it is not surprising that these correlations were significant in all clusters. More critically, the analyses confirmed the visual pattern in Fig. 2b: V_noise_ bias and AV_noise_ sensitivity were correlated with ERP_comp_ in the 500–540 ms window, and V_noise_ bias was also correlated with ERP_comp_ in the 590–630 ms window. To determine which of the components in the ERP_comp_ waveform drove the correlation with Acc_comp_, and thereby understand the neural basis of the behavioral effects, we correlated the amplitude difference-waves (relative to No_noise_) with Acc_comp_. For the temporal-spatial clusters in which ERP_comp_ was also correlated with bias and/or sensitivity, these correlations involved the residual ERP difference waves after regressing out the bias/sensitivity effect(s).

As illustrated in Fig. 3, the larger the difference between the No_noise_ and AV_noise_ ERPs in the 260–300 ms window, the larger Acc_comp_ was. In the 350–390 ms window, correlations in the same direction became significant for both unimodal noise ERP differences. These correlations were also observed in the 500–540 ms window, and the No_noise_ − A_noise_ ERP difference was also correlated with Acc_comp_ in the 590–630 ms window.

## Discussion

Across two experiments, we quantified noise compensation in simultaneously presented speech and print by subtracting the summed unimodal noise detriments relative to No_noise_ from the bimodal noise detriment relative to No_noise_. Because RT, bias, and sensitivity modulated accuracy, we assessed noise compensation after controlling for those variables, thereby providing a more rigorous treatment of noise compensation effects than in previous work (e.g., ref. 6).

The ERP data from Experiment 2 allowed us to disentangle processes related to noise compensation from processes related to bias and sensitivity at the neural level, and we observed that at 350–390 ms after stimulus onset, the ERP differences for the unimodal noise conditions relative to No_noise_ were correlated with Acc_comp_: the larger the ERP difference was, the larger Acc_comp_ was (as measured about 1500 ms later). The same correlations were observed in a 500–540 ms window, but during this time, the ERPs were also modulated by V_noise_ bias and AV_noise_ sensitivity. However, after regressing out the direct effect of bias and sensitivity on the ERP difference waves, the correlations with Acc_comp_ remained significant. As explained in the Supplementary Material, the timing of these effects aligns with an N400 effect that is usually associated with lexical/semantic processing, suggesting that noise compensation in audiovisual word processing occurs predominantly on a lexical/semantic level.

This is particularly interesting when considering that other research has demonstrated that integration of print and speech can occur on sub-lexical levels^15^,^16^. For example, effects of AV incongruence between simultaneously printed and spoken single letters takes place already at around 200 ms (i.e., when assessed in electrophysiological oddball paradigms, see refs 17 and 18. In the current study, we did observe positive correlations between the ERP_comp_ score and the Acc_comp_ score starting at around 200 ms, but these were not as robust as the later negative correlations. Moreover, the earliest time-window in which Acc_comp_ was correlated with an individual ERP difference wave was 260–300 ms, but since it involved the No_noise_ − AV_noise_ ERP difference (i.e., the larger the ERP difference, the larger noise compensation), and not the No_noise_ − A_noise_ or No_noise_ − V_noise_ differences, it is not directly interpretable as noise compensation. What, then, is the source of the discrepancy between our findings and previous work? One possibility is that by using full words, our task engages a different, potentially more natural/automatic set of audiovisual integration processes than single letter tasks, and this interaction occurs at a higher level of representation. A second possibility is that noise compensation is (partially) constituted on a sub-lexical level (see also refs 6 and 11). However, the effects of such compensation would then be fed forward (and potentially magnified) in higher order representations on lexical/semantic levels that are most relevant for the response system (although it is currently not exactly clear how and why response systems engage different levels of representation in different tasks, see ref. 19 for discussion).

A second set of highly relevant observations were that the correlations between Acc_comp_ and No_noise_ − A_noise_ accuracy were stronger than for No_noise_ − V_noise_ accuracy (see Table 1), and the correlations between the No_noise_ − A_noise_ ERP difference and Acc_comp_ were observed in more time-windows than the correlations between the No_noise_ − V_noise_ ERP difference and Acc_comp_. This suggests that print-driven noise compensation is more prominent than speech-driven compensation, and is consistent with similar asymmetric effects reported by Borowsky, Owen and Fonos^11^. In two related tasks, they presented participants with printed syllables in combination with noise-masked auditory syllables (e.g., /ta/), and asked participants to indicate what they had heard via a two-alternative-forced-choice response probe (e.g., *heard* /*ta*/ or *heard* /*da*/); or, they presented the auditory syllable in the clear, and the printed one in noise, and asked participants what they had seen. Critically, the syllables could be incongruent as well, and the authors observed that the facilitating effect of congruent print on noise-masked speech was larger than the cost induced by incongruent print. In contrast, the facilitation/cost of congruent/incongruent speech on noise-masked print was symmetrical. In a follow-up study with words rather than syllables^12^, the same pattern of results was observed and the authors therefore argued in favor of facilitation-dominant connections from orthography to phonology on both sublexical and lexical levels of processing, that outweigh connections from phonology to orthography.

A similar asymmetry is observed when the visual signal consists of an articulating mouth rather than print. A clear example is provided by past work in which auditory and visual speech are purposely selected to reflect the most ambiguous stimulus on the boundary of a phonetic or lip-read contrast (i.e. an ambiguous speech sound or lip-read video in between /aba/ and /ada/ that is perceived as either /aba/ or /ada/ in ~50% of trials). Repeated exposure to these ambiguous speech segments in combination with clear unambiguous input in the other modality has shown that the effect of unambiguous visual speech on the perception of ambiguous sounds (e.g., ref. 20) is larger than the effect of unambiguous auditory speech on ambiguous visual speech^21^. Clearly, visual speech and printed text do not engage identical neurocomputational circuits as auditory and visual speech. However, it is argued that mechanisms underlying AV speech integration for stimuli in which the visual input consists of print or visual speech are similar to some extent (e.g., ref. 22). A general asymmetry in cross-modal effects could therefore indicate that the system is biased towards compensating for auditory noise via visual speech (e.g., ref. 4) or print (e.g., ref. 6), and not vice versa due to the properties of domain-general noise compensation mechanisms.

Despite the fact that interactions between speech and print may occur on sub-lexical (e.g., refs 15 and 16) and lexical levels (e.g., ref. 12), our data suggest that noise compensation occurs mainly on a lexical/semantic level in the context of words. Auditory lexical processing is often observed before 350 ms 23,24,25,26,27,28, whereas we observed robust effects of noise compensation at 350–390 ms, and at 500–540 ms. Furthermore, the combined set of findings suggest a general primacy of visual stimuli in AV integration. This provides an intriguing direction for future research: Why would visual stimuli take precedence over auditory stimulation when constraint satisfaction is performed using cross-modal information? This is especially relevant if one considers the differences in acquisition trajectories of the different sources of information. That is, auditory and visual-speech information are both available early during development (e.g., two month old infants can already detect the correspondence between auditory and visual speech, ref. 29). In contrast, linguistic information conveyed by print becomes available only relatively late, with reading typically bootstrapping off the spoken language system.

The current paradigm, analytical method and findings can be used to conduct rigorous, direct comparisons of the time-courses that underlie cross-modal noise compensation for visual speech and print, and other analogous cross-modal integration processes (such as integration of speech and visual speech). This is particularly crucial because auditory and visual speech are tightly linked (e.g., the temporal characteristics are highly correlated) and biologically grounded through evolution, whereas this is not the case for the relationship between auditory speech and print. The present work therefore offers an innovative avenue for contrasting flexible domain-general vs. neurobiologically optimized domain-specific processing.

## Methods

### Experiment 1

#### Participants

Spanish adults (34 in total, 33 right-handed, 23 females, mean age = 21 years, SD = 2.3 years) participated in the experiment for payment. All participants had normal or corrected-to-normal vision and reported no language, hearing, or motor impairments. All participants used Spanish as their primary language and gave their informed consent prior to testing. The experiment was conducted in accordance with the Declaration of Helsinki and approved by the BCBL ethics committee.

#### Stimuli

The candidate stimulus population was determined using the EsPal subtitle database^30^ and was constrained using three criteria: word frequency (frequency ranged between 1 and 20 per million), word length (the number of phonemes ranged in between 3 and 10), and number of syllables (which was restricted to 2 or 3). Homophones (e.g., ‘vaca’ [cow], ‘baca’ [roof box]) were excluded, and 2659 words fulfilled the inclusion criteria. With the Stochastic Optimization of Stimuli (SOS) algorithm and software^31^, 400 pairs of words were identified (i.e., 800 words) in which the differences on the aforementioned criteria were minimal. For each pair, one word formed the basis for an audiovisually congruent stimulus, whereas the other was manually matched with a different word with similar psycholinguistic properties to form an incongruent stimulus. All stimuli were inspected by two native speakers who removed inflections, compound words, foreign words, and dialect-inappropriate words, which resulted in a final set of 304 test stimuli. The AV incongruent items were included for task purposes and not analyzed in detail (see also, ref. 32), but they were used to compute sensitivity and bias. A female native speaker of Spanish was recorded in a sound-proof booth while reading two differently ordered lists that included all items (i.e., each item was recorded twice). After cutting all items at on/offset, a native speaker of Spanish selected the recording of each stimulus that sounded most natural for use in the experiment.

#### Procedure

The experiment was run in a sound attenuated and dimly-lit booth. Participants were seated ~80 cm from a 48 cm (19-in) CRT monitor (100 Hz vertical refresh, 1024 px × 768 px resolution) on which the visual words were displayed in Arial font (font height was 5% of the display height, or ~38 px). Auditory words were delivered at a comfortable listening level through headphones.

In total, there were 608 trials (presented with PsychoPy, see ref. 33), with 152 trials per noise level (No_noise_, A_noise_, V_noise_ and AV_noise_). For each of the four noise levels, 76 trials were AV congruent, and 76 were incongruent. Visual noise was created by superimposing a rectangular field of 950 randomly positioned white dots (3 pixels in diameter) over an area slightly larger than the longest word in the stimulus set, and auditory noise was created by replacing 85% of the auditory waveform with signal-correlated noise.

**Figure 4:**
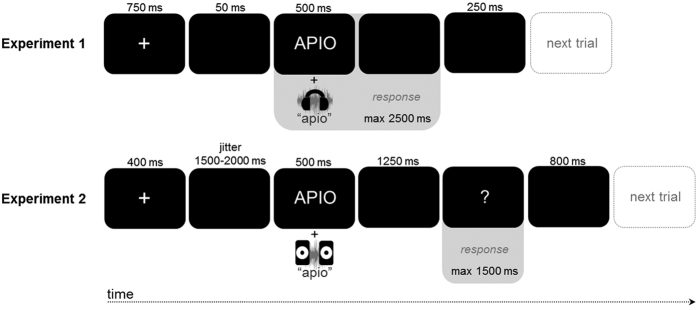
Trial Overview. When participants responded outside the response windows (indicated by the gray areas), they received a 2000 ms message on the screen stating that they should try to respond faster (Experiments 1 and 2) or later (Experiment 2), and such trials were excluded from analyses. A response within the window immediately prompted a 250 ms (Experiment 1) or 800 ms (Experiment 2) black screen.

Trials were distributed in random order across 17 blocks, with the first and last block containing 19 trials, and the 15 middle blocks containing 38 trials each. Blocks were separated by self-paced breaks. As shown in Fig. 4, each trial began with a fixation cross (750 ms), that was followed by a black screen (50 ms), after which the stimulus was delivered. Participants were asked to indicate whether speech and print matched or not by pressing the left or right CTRL key (as fast as possible, and within a 2500 ms window after stimulus onset) with the left or right index finger (“match” responses were indicated with the dominant hand). Once participants responded, the next trial began automatically after 250 ms. After each block, participants received feedback on the monitor regarding their accuracy and RT (based on correct responses) to keep them motivated. The experiment was preceded by a short practice session (10 trials), during which participants were acquainted with the task and procedure.

### Experiment 2

#### Participants

35 new right-handed Spanish adults with the same profile as those in Experiment 1 participated in return for payment. As described below, six participants were excluded because of substantial artifacts in the EEG signal. One participant was excluded because responses fell outside of the response time window (see Fig. 4) in 19% of trials. In the final set of 28 participants, there were 19 females, and mean age was 23 (SD = 2.3 years). All participants used Spanish as their primary language and gave their informed consent prior to testing. The experiment was conducted in accordance with the Declaration of Helsinki and approved by the BCBL ethics committee.

#### Stimuli and procedure

The stimuli were the same as in Experiment 1, but procedural details were tailored toward a non-speeded ERP paradigm: auditory words were delivered via two regular computer speakers (at ~65 dBA) placed on both sides of the monitor, and trial timing differed from experiment 1 (see Fig. 4).

#### EEG recording and analyses

The EEG was recorded at a 500 Hz sampling rate using a 32-channel BrainAmp system (Brain Products GmbH) and 28 Ag/AgCl electrodes that were placed in an EasyCap recording cap (electrode locations were Fp1, Fp2, F7, F3, Fz, F4, F8, FC5, FC1, FC2, FC6, T7, C3, Cz, C4, T8, CP5, CP1, CP2, CP6, P7, P3, Pz, P4, P8, O1, O2, and FCz [ground]). Four electrodes (2 on the orbital ridge above and below the right eye and 2 next to the lateral canthi of both eyes) recorded the vertical- and horizontal Electro-oculogram (EOG). Two additional electrodes were placed on the mastoid bones, of which the left was used to reference the signal on-line. Electrode impedance was adjusted to <5 kΩ (scalp electrodes) and <10 kΩ (EOG electrodes). Using Brain Vision Analyzer 2.0, the signal was re-referenced off-line to an average of the two mastoid electrodes and high-pass filtered (Butterworth zero phase filter at 24 dB/octave) at 0.5 Hz. Next, segments in the continuous EEG that contained course artifacts such as EMG bursts or glitches (defined as segments +/−100 ms around amplitude changes >60 μV/ms) were identified, and the signal was decomposed into independent components (i.e., ICA, e.g., ref. 34). The ICA decomposition (restricted infomax) was based on the entire data-set (not including the previously identified artifacts) and components that captured blinks or horizontal eye-movements (identified through visual inspection based on components’ energy and topography) were removed (the mean number of removed components was 3.32). Next, the data were low-pass filtered at 35 Hz (Butterworth zero phase filter at 24 dB/octave) and an additional 50 Hz notch filter was applied to remove residual electrical interference. The data were segmented into 1200 ms epochs (including 200 ms before, and 1000 ms after stimulus onset), and epochs that contained voltage steps >50 μV/ms, had a voltage difference >100 μV/1000 ms, minima/maxima <−100/>100 μV, and/or activity <0.5 μV were rejected. Six participants with a substantial artifact rate (mean = 36%) were excluded from analyses. For the remaining participants, averaged artifact rate was less than 15%. Epochs were base line corrected using the 200 ms of data before stimulus onset, averaged per condition, and the resulting ERPs were exported for statistical analyses.

## Additional Information

**How to cite this article**: Baart, M. *et al*. Cross-modal noise compensation in audiovisual words. *Sci. Rep.* **7**, 42055; doi: 10.1038/srep42055 (2017).

**Publisher's note:** Springer Nature remains neutral with regard to jurisdictional claims in published maps and institutional affiliations.

## Supplementary Material

Supplementary Information

## Figures and Tables

**Table 1:** Correlations between accuracy and bias (criterion c), accuracy and sensitivity (d′), and noise compensation (Acc_comp_) and accuracy differences of the noise conditions relative to No_noise_.

				r	p	r_p1_	p	r_p2_	p
Exp. 1	No_noise_ acc	×	No_noise_ c	−0.021	>0.250	−0.283	0.110	—	—
	A_noise_ acc	×	A_noise_ c	−0.364	0.034	−0.446	0.009	—	—
	V_noise_ acc	×	V_noise_ c	−0.292	0.094	−0.559	0.001	—	—
	AV_noise_ acc	×	AV_noise_ c	−0.464	0.006	−0.537	0.001	—	—
	No_noise_ acc	×	No_noise_ d′	0.860	<0.001	0.814	<0.001	—	—
	A_noise_ acc	×	A_noise_ d′	0.904	<0.001	0.821	<0.001	—	—
	V_noise_ acc	×	V_noise_ d′	0.811	<0.001	0.734	<0.001	—	—
	AV_noise_ acc	×	AV_noise_ d′	0.567	<0.001	0.577	<0.001	—	—
	Acc_comp_	×	No_noise_ − A_noise_ acc	−0.487	0.003	−0.512	0.004	−0.895	<0.001
	Acc_comp_	×	No_noise_ − V_noise_ acc	−0.545	0.001	−0.552	0.002	−0.686	<0.001
	Acc_comp_	×	No_noise_ − AV_noise_ acc	0.369	0.032	0.250	0.183	0.309	0.162
Exp. 2	No_noise_ acc	×	No_noise_ c	−0.771	<0.001	—	—	—	—
	A_noise_ acc	×	A_noise_ c	−0.828	<0.001	—	—	—	—
	V_noise_ acc	×	V_noise_ c	−0.559	0.002	—	—	—	—
	AV_noise_ acc	×	AV_noise_ c	−0.514	0.005	—	—	—	—
	No_noise_ acc	×	No_noise_ d′	0.635	<0.001	—	—	—	—
	A_noise_ acc	×	A_noise_ d′	0.672	<0.001	—	—	—	—
	V_noise_ acc	×	V_noise_ d′	0.569	0.002	—	—	—	—
	AV_noise_ acc	×	AV_noise_ d′	0.723	<0.001	—	—	—	—
	Acc_comp_	×	No_noise_ − A_noise_ acc	−0.425	0.024	—	—	−0.820	<0.001
	Acc_comp_	×	No_noise_ − V_noise_ acc	−0.415	0.028	—	—	−0.765	<0.001
	Acc_comp_	×	No_noise_ − AV_noise_ acc	−0.062	>0.250	—	—	−0.457	0.043

Correlation values r_p1_ are partial correlations controlled for RT (correlations involving Acc_comp_ were controlled for RT in all noise conditions). Correlation values r_p2_ are partial correlations that controlled for RT, bias and sensitivity in all conditions. Columns labeled ‘p’ represent the corresponding p-values.
