# Supplementary material for: Cross-modal noise compensation in audiovisual words
Source: Sci Rep. 2017 Feb 7;7:42055. doi: 10.1038/srep42055 (PMC5294401; doi:10.1038/srep42055)
Supplement: Supplementary Information [file srep42055-s1.pdf]

Supplementary Information

Cross-modal noise compensation in audiovisual words

Martijn Baart[1,2], Blair C. Armstrong[1,3], Clara D. Martin[1,4], Ram Frost[1,5,6], and Manuel Carreiras[1,4,7]

[1]*BCBL. Basque Center on Cognition, Brain and Language, Donostia - San Sebastián, Spain*

[2]*Department of Cognitive Neuropsychology, Tilburg University, Tilburg, the Netherlands*

[3]*Department of Psychology & Centre for French & Linguistics at Scarborough, University of Toronto, Toronto, Canada*

[4]*IKERBASQUE Basque Foundation for Science, Donostia - San Sebastián, Spain*

[5]*Department of Psychology, The Hebrew University of Jerusalem, Jerusalem, Israel*

[6]*Haskins Laboratories, New Haven, CT, USA*

[7]*University of the Basque Country. UPV/EHU, Bilbao, Spain.*

Figure S1 displays the ERPs for all noise conditions. Clearly, noise modulated the ERPs, as for instance indicated by the increased visual negative N1 and positive P2 at 100 ms and 200 ms respectively (see electrodes O1 and O2), for V_{noise} and AV_{noise} relative to No_{noise} and A_{noise}.

Figure S1. ERPs for all noise conditions time-locked to stimulus onset.

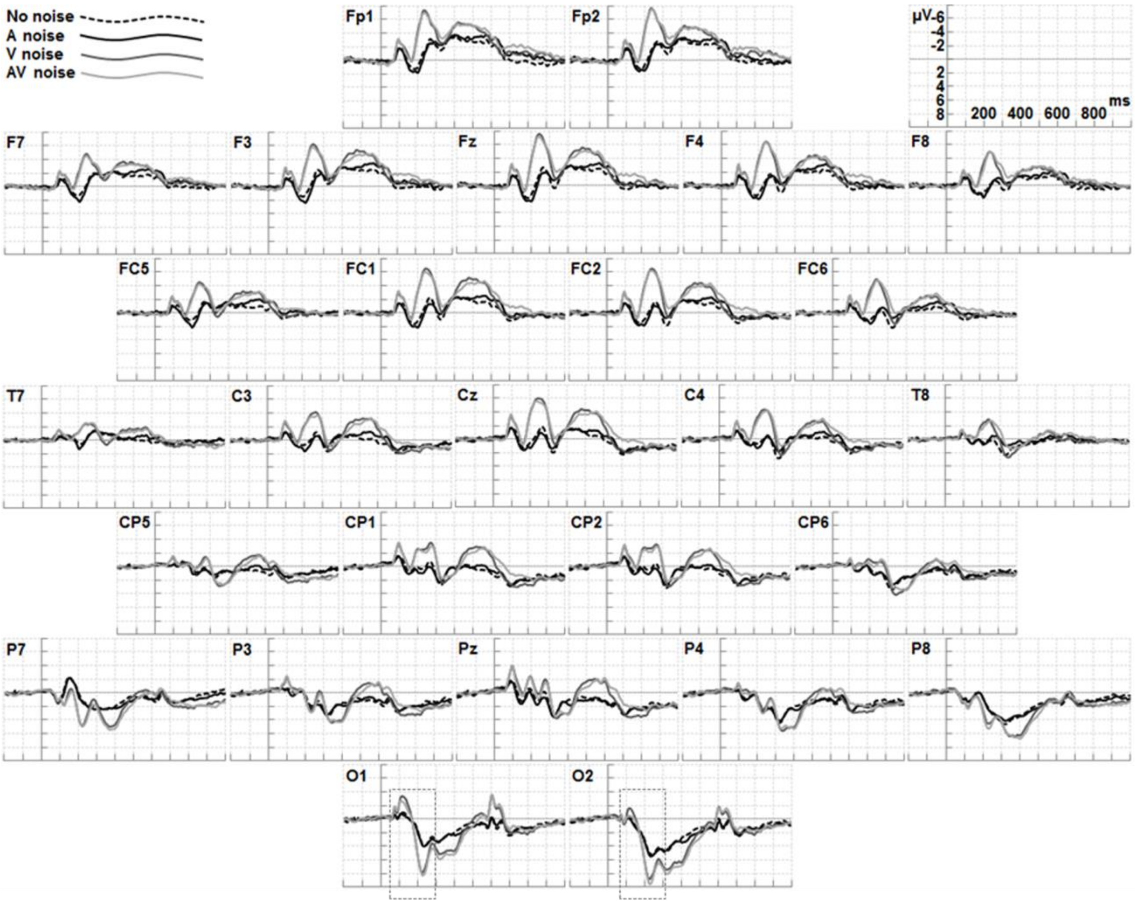

Given that the visual N1 is modulated by orienting attention to a relevant visual stimulus (1), such a finding is not surprising. However, it does indicate that directly comparing ERPs across noise-levels is not a particularly straightforward approach to assess cross-modal noise compensation because ERPs capture a variety of cognitive processes. Therefore, the main analyses focused on the (No_{noise} – AV_{noise}) – [(No_{noise} – A_{noise}) + (No_{noise} – V_{noise})] difference wave (ERP_{comp}), which we considered the ERP analogue of the Acc_{comp} score for accuracy.

These analyses, described in detail in the main text, revealed that the ERP_{comp} difference wave correlated with Acc_{comp} in several time-windows starting at 200 ms after stimulus onset, with the most prominent effects of noise compensation observed at 350-390 ms, and 500-540 ms, after controlling for response bias. That is, the No_{noise} - unimodal noise ERPs correlated with Acc_{comp} after regressing the direct effects of bias and sensitivity out of the ERPs. The timing of these effects coincides with the time-window of the N400, which is a negative deflection in the ERPs that occurs at around 200/300-600 ms post stimulus and is observed for spoken and written words whenever the stimulus deviates from what is expected (e.g.,2, 3, 4). The auditory and visual N400 effects may reflect similar processes, but the auditory N400 begins somewhat earlier and last longer than the visual N400 (e.g., 4). In general, the N400 is argued to reflect semantic integration (5) and lexical processing of spoken and written words is often found to precede the N400 (e.g.,6, 7, 8). However, the post-lexical nature of the N400 is disputed (e.g., 9) and we therefore deem it appropriate to characterize the N400 as reflecting lexical/semantic processes given that our stimuli were not aimed at disentangling these detailed distinctions. The overlap between the timing of our effects and the N400 therefore suggests that noise compensation was most prominent at the time-window associated with lexical/semantic processing, even though our ERP_{comp} difference wave does not perfectly reflect a typical N400 effect (which is not unexpected given how our task differs from the other cited tasks).

Our results are unlikely to be the result of a response conflict (i.e., when unimodal noise induces uncertainty about the matching or mismatching status of the stimulus), because the *larger* the accuracy detriment for A_{noise} or V_{noise} was relative to No_{noise}, the *smaller* the difference was between the No_{noise} and A_{noise} or V_{noise} ERPs. This trend was consistent in the 350-390 ms window, the 500-540 ms window, and the 590-

630 ms window. Although the correlations were never significant (*r*s in between 0 and -.232, *p*s > .093), they are in the opposite direction of what one would expect for a response conflict, where an *increase* in the accuracy difference (because the unimodal noise introduces uncertainty) should be related to an *increase* in ERP difference wave. That is, if one assumes that the larger the conflict is, the more negative the ERP will be (see for example 10, for a discussion on the Error Related Negativity and the N2), the difference between the No_{noise} (no conflict) and unimodal noise (high conflict) ERPs should increase with an increase in conflict, which is then likely to be reflected in an increase in the accuracy difference as well.

References

1. Luck, S. J., Heinze, H. J., Mangun, G. R., & Hillyard, S. A. Visual event-related potentials index focused attention within bilateral stimulus arrays. II. Functional dissociation of P1 and N1components. *Electroen. Clin. Neuro.* **75**(6), 528-542 (1990).

2. Brown, C., & Hagoort, P. The processing nature of the N400: Evidence from masked priming. *J. Cognitive Neurosci.* **5**(1), 34-44 (1993).

3. Holcomb, P. J., & Neville, H. J. Auditory and Visual Semantic Priming in Lexical Decision: A Comparison Using Event-related Brain Potentials. *Lang. Cognitive Proc.* **5**(4), 281-312 (1990).

4. Kutas, M., & Federmeier, K. D. Thirty years and counting: finding meaning in the N400 component of the event-related brain potential (ERP). *Annu. Rev. Psychol*. **62**, 621-647 (2011).

5. Hagoort, P. The fractionation of spoken language understanding by measuring electrical and magnetic brain signals. *Philos. T. R. Soc. B.* **363**(1493), 1055-1069 (2008).

6. Baart, M., & Samuel, A. G. (2015). Early processing of auditory lexical predictions revealed by ERPs. *Neurosci. Lett.* **585**, 98-102 (2015).

7. MacGregor, L. J., Pulvermüller, F., van Casteren, M., & Shtyrov, Y. Ultra-rapid access to words in the brain. *Nat. Commun.* **3**, 711; 10.1038/ncomms1715 (2012).

8. van den Brink, D., Brown, C. M., & Hagoort, P. Electrophysiological evidence for early contextual influences during spoken-word recognition: N200 versus N400 effects. *J. Cognitive Neurosci.* **13**(7), 967-985 (2001).

9. Deacon, D., Hewitt, S., Yang, C. M., & Nagata, M. Event-related potential indices of semantic priming using masked and unmasked words: evidence that the N400 does not reflect a post-lexical process. *Cognitive Brain Res.* **9**(2), 137-146 (2000).

10. Van Veen, V., & Carter, C. S. The anterior cingulate as a conflict monitor: fMRI and ERP studies. *Physiol. Behav.* **77**(4), 477-482 (2002).
